# Associations of Various Health-Ratings with Geriatric Giants, Mortality and Life Satisfaction in Older People

**DOI:** 10.1371/journal.pone.0163499

**Published:** 2016-09-22

**Authors:** Thomas Puvill, Jolanda Lindenberg, Jacobijn Gussekloo, Anton J. M. de Craen, Joris P. J. Slaets, Rudi G. J. Westendorp

**Affiliations:** 1 Leyden Academy on Vitality and Ageing, Leiden, the Netherlands; 2 Primary Care and Public Health, Leiden University Medical Center, Leiden, the Netherlands; 3 Gerontology and Geriatrics, Leiden University Medical Center, Leiden, the Netherlands; 4 Faculty of medical sciences, University of Groningen, Groningen, The Netherlands; 5 Department of Public Health and Center for Healthy Aging, Copenhagen University, Copenhagen, Denmark; Medizinische Universitat Innsbruck, AUSTRIA

## Abstract

Self-rated health is routinely used in research and practise among general populations. Older people, however, seem to change their health perceptions. To accurately understand these changed perceptions we therefore need to study the correlates of older people’s self-ratings. We examined self-rated, nurse-rated and physician-rated health’s association with common disabilities in older people (the geriatric giants), mortality hazard and life satisfaction. For this, we used an age-representative population of 501 participant aged 85 from a middle-sized city in the Netherlands: the Leiden 85-plus Study. Participants with severe cognitive dysfunction were excluded. Participants themselves provided health ratings, as well as a visiting physician and a research nurse. Visual acuity, hearing loss, mobility, stability, urinal and faecal incontinence, cognitive function and mood (depressive symptoms) were included as geriatric giants. Participants provided a score for life satisfaction and were followed up for vital status. Concordance of self-rated health with physician-rated (*k* = .3 [.0]) and nurse-rated health (*k* = .2 [.0]) was low. All three ratings were associated with the geriatric giants except for hearing loss (all *p* < 0.001). Associations were equal in strength, except for depressive symptoms, which showed a stronger association with self-rated health (.8 [.1] versus .4 [.1]). Self-rated health predicted mortality less well than the other ratings. Self-rated health related stronger to life satisfaction than physician’s and nurse’s ratings. We conclude that professionals’ health ratings are more reflective of physical health whereas self-rated health reflects more the older person’s mental health, but all three health ratings are useful in research.

## Introduction

Self-rated health has captured the interest of researchers for over half a century [1], mostly because it has consistently been associated with a variety of health indicators [2] and longevity [3, 4]. Therewith, it appears to reflect the state of the body to a large extent, arguably even capturing some aspects of health that objective health indicators do not.

It is uncertain if the properties attributed to self-rated health still hold for older people. Qualitative and quantitative research suggests that older people appraise their health differently, as they realise that disease and disability are more common at old age [5, 6]. Indeed, the association between a wide range of physical health indicators and self-reported health becomes smaller with age [7]. At the same time, the association between mental health and self-rated health grows stronger [8, 9]. Not only older people themselves, but also physicians and other healthcare professionals struggle with questions of normativity regarding the health of their older patients. Learning to which extent these ratings reflect the state of physical health and how they are associated with older people’s mortality hazard and life satisfaction is necessary in order to aptly understand self-rated health and health-ratings of healthcare professional, and to determine these measures’ usefulness as a prognostic and a research instrument, at an age when from a biomedical perspective nearly no-one is healthy anymore.

The current study investigates and compares the correlates of self-rated health with physical and mental health in a representative population of 85-year olds from the Netherlands. We study the different associations of self-rated health with i) geriatric giants, ii) mortality hazard and iii) life satisfaction. To explore the value of how health is viewed by professionals, we investigate the same associations with the health ratings of a visiting physician and a research nurse.

## Methods

### Subjects

All inhabitants of the municipality of Leiden, the Netherlands, who reached age 85 in the period of September 1997 and September 1999 (n = 705) were identified from the Dutch municipal central registry for this study and invited to participate shortly after their 85^th^ birthday. 14 Persons passed away before enrolment, 92 declined participation, and all 599 remaining individuals (87% of the invited population) participated [10]. For the current analysis, those with severe cognitive dysfunction, operationalised as MMSE score < 19 points, were excluded. This concerned 98 participants, setting the final study sample at 501. Information on sex and marital status was collected from the municipality. Participants themselves provided information on their income, education and living situation.

### Measures and Procedure

Participants received home visits by one out of two trained physicians and later by a trained research nurse shortly after inclusion. The physicians had just finished medical school. The nurse was an experienced district nurse. During the home visits, various interviews and physiological measurements were taken. There were no encounters between the healthcare professionals and the older people other than the home visits.

For self-rated health, participants were asked ‘How would you rate your health in general?’ Answer categories were ‘poor’, ‘moderate’, ‘good’, ‘very good’ and ‘excellent’. The last two categories were combined to make these self-rated health ratings comparable with physician- and nurse-ratings of health. One out of two physicians and a research nurse who had visited all the participants provided ratings of health. At the end of the visit, they were asked: ‘How would you rate the health of this older person in general?’ Answer categories were ‘poor’, ‘moderate’, ‘good’ and ‘very good or excellent’.

The geriatric giants, often-occurring functional impairments in older people that delineate core areas in geriatrics, are included as physical health indicators[11]. As additions to the list original list of geriatric giants are commonly made [12], we added depressive symptoms and sensory impairment that also fit the description of geriatric giants. Visual acuity was measured with a high-contrast portable letter chart [13]. Distance to the chart was three meters; light level was standardized at 500 lux. Wearing of visual aids was permitted. Hearing loss was measured with an audiological test using pure-tone audiometry [14]. Hearing loss was estimated by the Fletcher index: the best ear’s hearing loss in dB, averaged over 1000, 2000 and 4000 Hz. Hearing loss was assessed on a separate day after the participants were visited by the research physician and nurse. This led to a 21% non-response for audiometry. For those who did not participate, we replaced the medianised objective hearing loss test by a medianised subjective hearing loss composite rating that consisted for two-third of hearing evaluations provided by the physician and the nurse, and for one-third by the participant’s self-reported ability to follow a two-way and a four-way conversation. Cognitive function was measured with the MMSE [13]. For the analyses, the median was used as a cut-off as the most severely cognitively impaired individuals were already excluded. Mobility was measured as gait speed in seconds during the first 3 meters of a standardised walking test [13]. Stability was measured as speed in seconds to complete the first two repetitions of a standardised stand-up test [15]. For both speed and stability, median speed was calculated separately for both sexes and participants who did not complete the task were categorised as below the median. Mood was measured as depressive symptoms with the Geriatric Depression Scale, using a cut-off of 5 points or above [16]. All outcomes were dichotomised on the sample’s median and one point was awarded for every health indicator, the sum score used for analysis. Urinal and faecal incontinence was coded as present or absent, and the depressive symptoms screening instrument below or above the cut-off for depression.

Mortality hazard was determined by the participants’ number of remaining years after age 85 using vital status in the municipal registry. This information was last collected February 1^st^ 2014. As all participants were followed for vital status, none were last to follow-up.

To measure life satisfaction, participants were presented with Cantril’s ladder [17]: a depiction of a ladder, the steps numbered one to ten. Participants were asked to imagine that the bottom step is the worst and the top step is the best possible life for them, and to then indicate where on the ladder they felt they stood at that time. The number that corresponded with the step was the participant’s life satisfaction score.

Mobility, stand-up speed, cognition and mood were ascertained during the physicians’ home visit. Incontinence, visual acuity, self-rated health and life satisfaction were ascertained during the nurse’s home visit.

### Data Analysis

The agreement between various ratings of health was determined with Cohen’s Kappa using SPSS Statistics 21 for Windows.

The associations between the geriatric giants and self-, physician- and nurse-rated health were estimated with multiple regression analysis, adjusting for sex, marital status, income, education and living situation. The analyses were performed with the number of geriatric giants and additionally with each geriatric giant in separate analyses. All health scorings were given a number from one to four when entered as a continuous variable in the analyses and were performed with robust standard errors due to possible violation of the assumptions of heteroscedasticity in some of the analyses [18].

To study predictors of mortality hazard at age 85, we used Cox-regression analyses. Visual inspection of the cumulative hazard curve showed the assumption of proportional hazard was met for self- and nurse-rated health. The curve for physician-rated health was not perfect, but showed no cause for concern. Separate sets of analyses were performed with self-rated health, with physician-rated health and with nurse-rated health as predictors: once crude, once adjusting for demographic characteristics and once adjusting for demographic characteristics and all geriatric giants. Because observations in the category ‘poor’ were scarce, health ratings were dichotomised by merging response categories ‘poor’ and ‘moderate’ and categories ‘good’ and ‘very good or excellent’. The cox regression model that included self-rated health was compared with those that included physician-rated and nurse-rated health using the likelihood ratio tests using 1 degree of freedom.

Finally, the association between life satisfaction and self-, physician- and nurse ratings of health was estimated with multiple regression analysis with robust standard errors, adjusting for sex, marital status, income, education and living situation.

For nurse-rated health, life satisfaction, visual acuity and hearing loss after replacement, around 5% of data were missing. For all other variables, no more than 1% was missing. Missing values were imputed with multiple imputation based on all variables in this study as predictors, using predictive mean matching. Imputations were done with the full-range variables. 100 Imputations were used to ensure that estimates of the variability of the imputations were adequate [19].

### Ethics

The Medical Ethical Committee of the Leiden University Medical Center approved of the study. Permission to participate was provided in writing by participants. When the participant was unable to give permission, permission was provided by a guardian.

## Results

### Description of the Population and Health Ratings

Table 1 shows characteristics of the subjects under study. The participants were comparable to the entire research sample and to the source population in sex, marital status, income, education level and mortality hazard [10]. Table 1 further shows that more than 70% of all health ratings was good or excellent. Five participants are still alive at the time of writing.

**Table 1:** Study Population Characteristics at Age 85.

Characteristics	N = 501
Demographics	
Male / female (%)	184 (36.7%) / 317 (63.3%)
Married / not married (%)	176 (35.1%) / 325 (64.9%)
Additional income / state pension only (%)	430 (85.8%) / 71 (14.2%)
Further education / primary school only (%)	197 (39.3%) / 304 (60.7%)
Living independently / institutionalised (%)	446 (89%) / 55 (11%)
Geriatric giants	
Hearing loss in dB (median, IQR)	50 (38–57)
Visual acuity (median, IQR)	.6 (.5 –.8)
Walking test performance in seconds (median, IQR)	
Women	4.0 (3.2–5.8)
Men	3.6 (2.8–4.6)
Stand up test performance in seconds (median, IQR)	
Women	4.6 (3.5–6.3)
Men	4.2 (3.3–5.6)
Incontinent / continent (%)	168 (33.5%) / 333 (66.5%)
MMSE score (median, IQR)	27 (24–28)
Depressive symptoms^a^ present/absent (%)	78 (15.6%)/433 (84.6%)
Number of geriatric giants	
None (%)	25 (5.0%)
1 (%)	64 (12.8%)
2 (%)	123 (24.6%)
3 (%)	99 (19.8%)
4 (%)	74 (14.8%)
5 (%)	75 (15.0%)
6 (%)	28 (5.6%)
7 (%)	13 (2.6%)
Health ratings	
Self-rated health	
Poor or moderate / good or excellent (%)	147 (29.3%) / 354 (70.7%)
Physician-rated health	
Poor or moderate / good or excellent (%)	115 (23.0%) / 386 (77.0%)
Nurse-rated health	
Poor or moderate / good or excellent (%)	145 (28.9%) / 356 (71.1%)
Life satisfaction	
Life satisfaction score (median, IQR)	8 (7–9)
Mortality hazard	
Mortality hazard at age 85 in years (median, IQR)	6 (4–10)

^a^ Defined as a GDS score of 5 or above (see methods)

We compared estimations of self-rated health with the physician and nurse ratings using cross-tabulations (Table 2). For 53% (*k* = .3 [.0]) of the persons the physician-rated health was concordant with the self-rated health and this was 50% (*k* = .2 [.0]) for the nurse-rated health compared to the self-rated health.

**Table 2:** Discrepancy between Self-rated Health and Physician-rated and Nurse-rated Health.

		**Self-rated health**				
		Poor	Moderate	Good	Very good	Total
**Physician-rated health**	Poor	3	0	3	0	6
	Moderate	12	53	41	3	109
	Good	6	70	158	63	297
	Very good	0	3	33	53	89
	Total	21	126	235	119	501
		Poor	Moderate	Good	Very good	Total
**Nurse-rated health**	Poor	1	2	1	0	4
	Moderate	16	66	49	10	141
	Good	4	54	148	72	278
	Very good	0	4	37	37	78
	Total	21	126	235	119	501

Cells represent number of participants.

### Health Ratings and the Geriatric Giants

**Fig 1:**
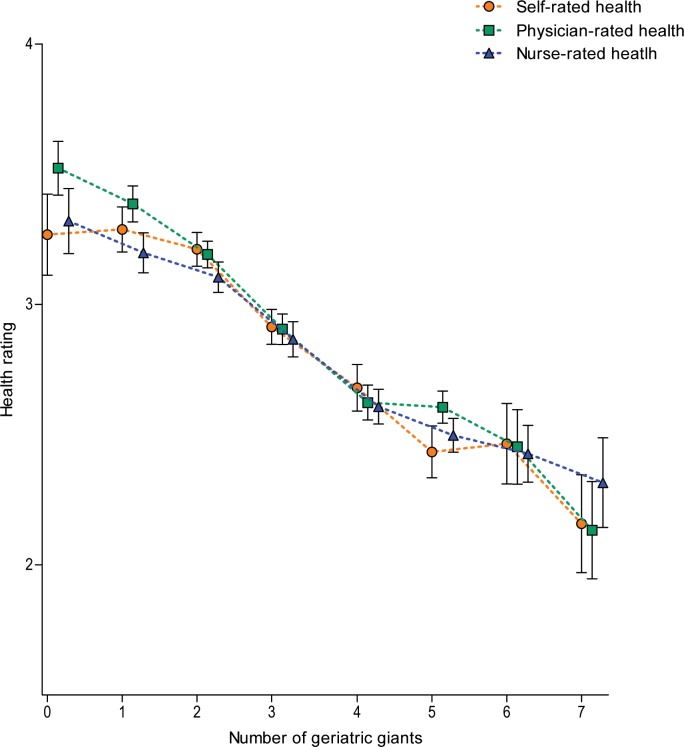
Mean self-, physician- and nurse-rated health predicted by the number of geriatric giants. Estimates by regression analysis with robust standard errors adjusted for demographic characteristics.

Fig 1 shows estimates of self-rated health by the number of geriatric giants. The estimates of physician- and nurse-rated health are also depicted. A higher geriatric giants composite score was strongly associated with worse self-, physician- and nurse-rated health (trend analysis, *p* < .001 for all).

Table 3 shows the estimates of the differences in self-rated health depending on demographic variables and the separate geriatric giants, as well as for physician- and nurse-rated health. All geriatric giants except hearing loss were associated with lower self-, physician- and nurse-rated health (all *p* < 0.001). Having depressive symptoms was most strongly associated with lower self-rated health -.8 (SE .1) points (*p* < .001) and this was significantly stronger than physician- and nurse-rated health -.4 (SE .1) points (*p* < .001 for both). Beside depressive symptoms, none of the associations between self-rated health and physician- or nurse-rated health with the demographic variables or geriatric giants were statistically significantly different from each other.

**Table 3:** Differences in Self-, Physician- and Nurse-rated Health, Dependent on Demographic and Health Variables.

	Self-rated health		Physician-rated health		Nurse-rated health	
Characteristics	Points (SE)	*P*	Points (SE)	*P*	Points (SE)	*p*
Demographic						
Male vs female	-.1 (.1)	.534	-.0 (.1)	.691	-.2 (.1)	.003
Unmarried vs married	-.0 (.1)	.646	-.0 (.1)	.657	.0 (.1)	.681
State pension only vs additional income	-.1 (.1)	.621	-.1 (.1)	.245	-.1 (.1)	.176
Primary school only vs further education	-.1 (.1)	.400	-.2 (.1)	.003	-.2 (.1)	.003
Institutionalised vs living independently	-.6 (.1)	< .001	-.6 (.1)	< .001	-.4 (.1)	< .001
Geriatric Giants						
Sensory						
Worse vs better visual acuity	-.2 (.1)	.002	-.2 (.1)	< .001	-.2 (.1)	.001
More vs less hearing loss	.1 (.1)	.312	-.0 (.1)	.328	.0 (.1)	.710
Mobility						
Slower vs faster walking test speed	-.5 (.1)	< .001	-.4 (.1)	< .001	-.4 (.1)	< .001
Balance						
Slower vs faster stand-up speed	-.4 (.1)	< .001	-.5 (.1)	< .001	-.4 (.1)	< .001
Incontinence						
Incontinent vs continent	-.2 (.1)	.003	-.3 (.1)	< .001	-.2 (.1)	< .001
Cognitive function						
Lower vs higher cognitive function	-.2 (.1)	.005	-.2 (.1)	.003	-.2 (.1)	< .001
Mood						
More vs less depressive symptoms	-.8 (.1)	< .001	-.4 (.1)	< .001	-.4 (.1)	< .001

Estimated by regression analyses with robust standard errors. Health ratings ranged from one to four. Adjusted for sex, marital status, income, education and living situation.

### Health Ratings and Mortality Hazard

Table 4 shows hazard ratios for mortality by self-, physician- and nurse-rated health. In the crude analysis, self-, physician- and nurse-rated health were all significant predictors of mortality hazard (all *p* < .001). After adjusting for demographic variables and presence of geriatric giants, the relation between physician- and nurse-rated health and mortality hazard became somewhat smaller, but remained significant (The confidence interval for the mortality hazard ratio of physician-rated health was 1.017–1.627; we rounded the lower bound off downwards in the table). The relation between self-rated health and mortality hazard became insignificant (hazard ratio 1.2, 95% CI 1.0–1.5, *p* = .068).

**Table 4:** Hazard Ratios for Mortality at Age 85, Dependent on Self-, Physician- and Nurse-rated Health.

	Poor to moderate self-rated health^a^		Poor to moderate physician-rated health^a^		Poor to moderate nurse-rated health^a^	
	Hazard Ratio	*p*	Hazard Ratio	*p*	Hazard Ratio	*p*
Crude	1.5 (1.2–1.8)	< .001	1.6 (1.4–1.8)	< .001	1.8 (1.5–2.2)	< .001
Adjusted for demographic variables	1.4 (1.3–1.6)	.001	1.5 (1.4–1.7)	< .001	1.7 (1.4–2.1)	< .001
Adjusted for demographic variables and geriatric giants	1.2 (1.0–1.5)	.068	1.3 (1.0–1.6)	.040	1.5 (1.2–1.9)	< .001

Estimates by Cox regression.

^a^ Compared to good, very good or excellent self-rated health.

We compared the Cox regression model that included self-rated health with those that included physician-rated and nurse-rated health. The crude model for physician ratings (*p* < .05) and all models for nurse-rated health (*p* < .01) had a significantly better model fit than the corresponding model for self-rated health.

### Health Ratings and Life Satisfaction

**Fig 2:**
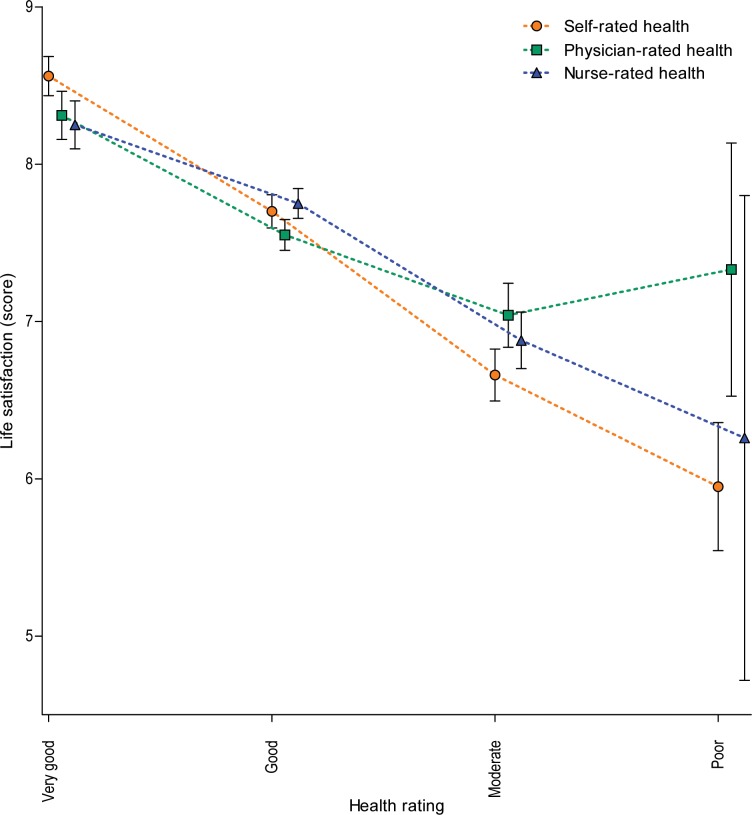
Mean life satisfaction scores at age 85, by self-, physician- and nurse-rated health. Estimates with regression analysis with robust standard errors adjusted for demographic characteristics.

Fig 2 shows self-reported life satisfaction dependent on scores of self-, physician- and nurse-rated health. Decrease in life satisfaction was .8 (SE .1) point with each lower category of self-rated health, whereas it was .5 (SE .1) points for physician-rated health and .7 (SE .1) for nurse-rated health (adjusted for demographics, all *p* < .001). Coefficients of determination for the models (adjusted R^2^) indicated that there was respectively 17.2%, 4.6% and 7.5% explained variance for the associations between self-, physician- and nurse-rated health and life satisfaction.

## Discussion

The findings of the present analyses are several fold. First, there are substantial differences between self-, physician and nurse-rated health. Second, all three of the health ratings investigated were associated with all geriatric giants with the exception of hearing loss. Third, we found that depressive symptoms had the strongest impact on self-rated health. Fourth, the health ratings of older people themselves predicted mortality hazard less well than professionals health ratings did, Finally, fifth, we established that self-rated health, instead, was most strongly associated with life satisfaction. Taken together, these findings show that health ratings are useful in research and clinical practise as a reflection of older people’s physical state and wellbeing, although each of the various health ratings point to different aspects of health. The professionals’ rated health score is more reflective of physical health whereas self-rated health reflects more the older person’s mental health. Perhaps older people and healthcare professionals emphasize different aspects of health in their ratings.

The dissociation between hearing loss and all three health ratings seems perhaps surprising, as the found median hearing loss of 50 dB is far above the Dutch cut-off of 35 dB for severe hearing impairment. However, in a sub-study of the Leiden 85-plus population, it was found that more than half out of those with objectively measured severe hearing impairment did not consider themselves severely impaired in hearing [20]. Other studies have also failed to find a consistent association between hearing loss and functional status [21, 22] and mental health [23, 24].

As already shown previously in the literature [3, 25, 26], agreement between self-rated and healthcare professional-rated health was low. Yet, the geriatric giants–a range of common health and or functional problems at old age—are grossly equally correlated with all three health ratings in this study. These seemingly paradoxical findings can perhaps be explained by the process of calibration in a single-item evaluation, as is done for multifaceted constructs such as health. In choosing a rating, individuals weigh the different aspects of their health. Differences in this weighing process can be absent on a group level, but nevertheless be found on the individual level when we directly compare the ratings.

The current study shows that depressive symptoms are more strongly associated with self-rated health than with physician-rated and nurse-rated health. Apparently, older people attach greater value to their mood than health professionals when rating health. This stronger role of mood in self-rated health could be specific for older people, as earlier research showed that mental health becomes increasingly associated with self-rated health over time, whereas physical health shows a decreasing association with self-rated health [2, 7, 8].

Particularly striking is that while self-rated health was still associated with mortality hazard, the association was no longer significant after correcting for the demographic variables and the geriatric giants. Though the *p*-value was still close to .05, this is a notable contrast to a bulk of studies that show that in the general population, self-rated health is a strong predictor of mortality, even after adjusting for physical health indicators [3, 4]. This may be, in line with the found stronger association with depressive symptoms, because older individuals hold a weaker emphasis on physical aspects of health than younger individuals [2, 7, 8]. Finally, it was shown that self-rated health was less associated with mortality than healthcare professionals’ ratings, suggesting that the healthcare professional is more focused on physical health when providing health ratings than the older individual.

The strong association between self-rated health and life satisfaction has been found in previous studies [27, 28]. Qualitative interviews conducted with a subgroup of our participants revealed that in order to cope with their declining health, older people normalise their health status by shifting their view on what good health entails [6]. The weaker association of self-rated health with mortality hazard could be indicative of this reframing of health, whereas the stronger associations between mental health and self-rated health and self-rated health and life satisfaction is perhaps indicative of older people’s redefinition of health. Alternatively, it could be evidence that this redefining is adaptive for older people.

### Implications

The current findings provide medical professionals with knowledge on what self-, physician- and nurse-rated health reflect in geriatric research. It gives them an estimate of the prognostic value of asking older individuals about their general health. The answer to the general health question is strongly associated with one’s overall mood and low health evaluations could be indicative of psychosocial problems. Although older people’s self-rated health still predicts mortality hazard, this association is much less pronounced than at younger ages.

### Strength and Weaknesses

Although a number of studies have already focussed on correlates of self-rated health at old age, few of these studies have compared their ratings to how healthcare professionals rate their health. We show in a representative sample that older people, despite having the same components of health in view, differently appreciate how these elements impact the way they perceive their overall health status than healthcare professionals do. We also show that the association between self-rated health and mortality after adjusting for the geriatric giants is small, in striking contrast to this association at other ages.

Another strength of current study is that we included the most common health problems at old age across various domains, i.e. geriatric giants, which is necessary for an adequate investigation of the associations between health status and global health ratings. Furthermore, the geriatric giants were mostly objectively measured, except for incontinence.

A further strength is that only one out of two physicians and one nurse rated the health status of all older people, which minimises the risk of variance between the professional raters. At the same time, using a small number of raters means that it is uncertain whether these results can be extrapolated to other healthcare professionals, though some confidence can be gained from the fact that our findings do not contradict earlier studies on the relationship between physician-rated health and physical and mental health [26, 29], self-rated health [25, 26] and mortality hazard [30]. Another factor, which may limit generalisability of these healthcare professionals’ ratings to the contemporary situation, is that the baseline data for current study were collected between 1997 and 1999. Since then, major changes have taken place in medical care. One important aspect in this is that in the meantime, patient-centred care has gained ground in the medical domain [31], which might have brought health ratings of healthcare professionals and patients closer together than shown in current study. Furthermore, when rating the older people’s health, the information that the physicians and nurse had at their disposal from having administered the research is not comparable with the information that a healthcare professional has at his or her disposal in clinical practise.

A weakness of current study is that it contained a large amount of missing data for audiometry. However, we were able to replace the missing audiometry data with subjective hearing ratings. As we included self-report, physician-report and nurse-report equally in these ratings, it is unlikely that this biased the association with self-, physician- and nurse-rated health.

The physician and nurse had performed different measurements and therefore had different information available on which they could base their judgement, but we note that still correlates between health ratings and all geriatric giants were very similar. Also, the overlap between self-rated health and nurse-rated health was smaller than between self-rated health and physician-rated health, although the nurse had asked the older people to rate their health. This makes us question to what extent the specific information obtained from the measures played a large role in rating the older person’s health. Although the nurse and physicians differed in the amount of clinical experience they had, a comparison of that falls beyond the scope of this paper as the objective of the paper was not to compare the nurse’s and physicians’ rating with each other, but with self-rated health.

### Future Research

Future research could compare self-rated and professional-rated health using more recent data, as current developments in patient-centred care may have brought the physician’s ratings of health closer to self-rated health.
